# DPHD from *Curcuma comosa* Enhances the Expansion and Osteogenic Differentiation of Human Umbilical Cord-Derived Mesenchymal Stem Cells While Attenuating Senescence

**DOI:** 10.1021/acsomega.5c08638

**Published:** 2026-05-18

**Authors:** Nittaya Boonmuen, Moe Moe Paing, Nareerat Sutjarit, Pakpoom Kheolamai, Sirikul Manochantr, Chairat Tantrawatpan, Waraluck Chaichompoo, Apichart Suksamrarn, Duangrat Tantikanlayaporn

**Affiliations:** † Department of Physiology, Faculty of Science, 68000 Mahidol University, Bangkok 10400, Thailand; ‡ Center of Excellence in Stem Research and Innovation, Faculty of Medicine, 37699 Thammasat University, Pathum Thani 12120, Thailand; § Nutrition Unit, Faculty of Medicine Ramathibodi Hospital, 26687 Mahidol University, Bangkok 10400, Thailand; ∥ Division of Cell Biology, Faculty of Medicine, Thammasat University, Pathum Thani 12120, Thailand; ⊥ Department of Chemistry and Center of Excellence for Innovation in Chemistry, Faculty of Science, 54780 Ramkhamhaeng University, Bangkok 10240, Thailand

## Abstract

Osteoporosis and other degenerative bone disorders are major health burdens, and current therapies are limited in restoring bone regeneration. Human umbilical cord-derived mesenchymal stem cells (hUC-MSCs) offer promise for regenerative medicine, but their clinical potential is restricted by replicative senescence during ex vivo expansion. In this study, we examined the effects of (3*R*)-1,7-diphenyl-(4*E*,6*E*)-4,6-heptadien-3-ol (DPHD), a diarylheptanoid derivative from *Curcuma comosa* Roxb, on hUC-MSC proliferation, senescence, migration, and osteogenic differentiation. Treatment with DPHD (2.5–5 μM) enhanced cell viability and proliferation while preserving MSC-specific marker expression and stemness-related genes (*NANOG*, *OCT4*, *SOX2*). DPHD significantly reduced senescence-associated β-galactosidase activity and reduced the expression levels of the cell cycle regulators p16 and p21, indicating an antisenescent effect. Moreover, DPHD suppressed the secretion of cytokines associated with the Senescence-Associated Secretory Phenotype (SASP), including IL-1β, IL-6, and MCP-1, and attenuated H_2_O_2_ induced ROS accumulation, confirming its antioxidant property. Furthermore, DPHD promoted osteogenic differentiation, as shown by increased ALP activity, calcium deposition, and upregulation of osteogenic marker genes. Mechanistically, DPHD upregulated Wnt/β-catenin-associated genes during proliferation (*CTNNB1*, *C-MYC*, *AXIN2*, *CCND1*) and enhanced osteogenic gene expression during differentiation (*RUNX2*, *ALP*, *COL1A1*), while inhibition of Wnt/β-catenin signaling suppressed these increases. Therefore, both the proliferative and osteogenic effects of DPHD are at least partly mediated through activation of the Wnt/β-catenin pathway. Collectively, these findings suggest that DPHD enhances the expansion and osteogenic potential of hUC-MSCs by attenuating cellular senescence while preserving their functional properties, underscoring its potential as a natural compound for improving MSC-based therapies in regenerative medicine.

## Graphical abstract

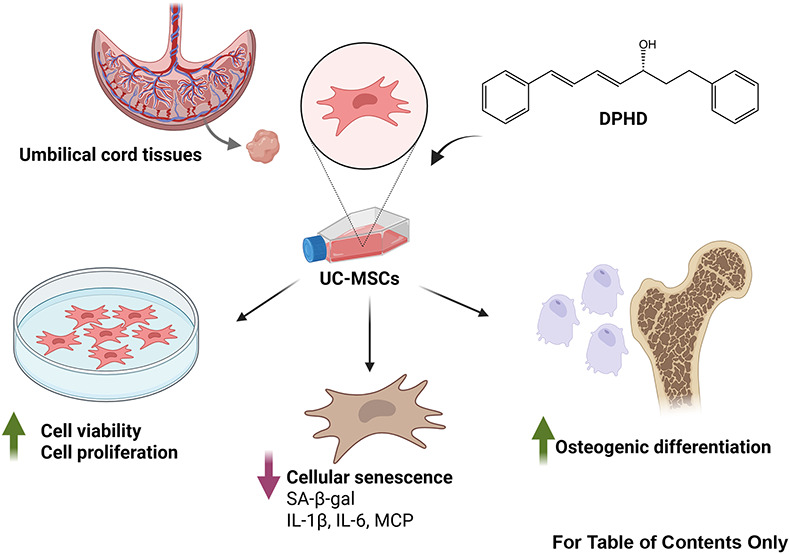

## Introduction

Osteoporosis, a chronic skeletal disorder decreased bone mass and deterioration of bone microarchitecture, represents a major global health concern, particularly in aging populations.^1^ It increases the risk of fragility fractures and impaired bone healing, leading to significant morbidity and socioeconomic burden. Although current therapies, such as anabolic and antiresorptive drugs, can slow disease progression, they are often limited by side effects and reduced long-term efficacy.^2^ Therefore, alternative regenerative strategies are urgently needed to restore bone mass and enhance skeletal repair.

Mesenchymal stem cells (MSCs) have emerged as promising candidates for bone regenerative therapy due to their multipotent capacity to differentiate into osteoblasts, adipocytes, and chondrocytes.^3,4^ Among the various sources of MSCs, human umbilical cord-derived MSCs (UC-MSCs) are particularly attractive because of their high proliferative potential, low immunogenicity, and noninvasive procurement.^5^ Compared with bone marrow-derived MSCs, UC-MSCs demonstrate greater expansion capacity and more stable phenotypes, rendering them suitable for bone tissue engineering and for the treatment of degenerative skeletal conditions such as osteoporosis and delayed fracture healing.^6^ However, the therapeutic efficacy of MSC-based bone regeneration is often hampered by a progressive decline in proliferation and differentiation capacity during vitro expansion.^7,8^ Senescent MSCs exhibit morphological alterations, reduced self-renewal, impaired immunomodulatory function, and diminished osteogenic differentiation.^9^ Moreover, senescence-associated secretory phenotype (SASP) factors, including pro-inflammatory cytokines, further impair the regenerative microenvironment, thereby reducing the clinical potential of MSCs.^10^ Strategies that can alleviate senescence, preserve stemness, and maintain differentiation potential are therefore critical to maximize the therapeutic utility of MSCs.

In this context, natural bioactive compounds have gained considerable attention as potential enhancers of MSC proliferation and differentiation.^11^ Among them, diarylheptanoids derived from *Curcuma comosa* Roxb are of particular interest. DPHD (1,7-diphenyl-(4*E*,6*E*)-4,6-heptadien-3-ol) is a polyphenolic phytochemical traditionally used in Southeast Asia for women’s health, anti-inflammatory therapy, and bone support.^12,13^ Pharmacological evidence indicates that DPHD exerts multiple bioactivities, such as antioxidative, anti-inflammatory, estrogenic, and osteoprotective actions.^14^ More recently, DPHD has been shown to stimulate proliferation and osteogenic activity in human osteoblasts.^15^ Importantly, DPHD exerts these osteogenic effects, at least in part, through activation of the Wnt/β-catenin signaling pathway, which is a critical regulator of the proliferation of MSCs and their osteogenic differentiation.^16^ Interestingly, recent studies have reported that DPHD also enhances ex vivo expansion and preserves the functionality of hematopoietic stem cells.^17^ These properties make DPHD an interesting candidate to counteract senescence and enhance the functional performance of MSCs in regenerative medicine.

In this study, we examined the effects of DPHD on the in vitro expansion and osteogenic differentiation of human UC-MSCs. We specifically assessed its influence on cell viability, proliferation, senescence, inflammatory cytokine secretion, and osteogenic differentiation, with the aim of enhancing the therapeutic utility of UC-MSCs for osteoporosis and other degenerative bone disorders. To our knowledge, this is the first report to demonstrate the direct effects of DPHD on UC-MSCs, providing new insights into its potential as a natural adjunct for MSC-based bone tissue engineering and regenerative medicine.

## Materials and Methods

### Ethics Approval and Consent to Participate

All procedures involving human umbilical cord tissue, including the donor consent process, were approved by the Human Research Ethics Committee, Faculty of Medicine, Thammasat University (COA 146/2021; MTU-EC-DS-6-067/64; approval date: June 21, 2021). The research was carried out in accordance with the ethical principles of the Declaration of Helsinki, the Belmont Report, and ICH-GCP, and complied with applicable rules and legislation. Before sample collection, all donors received written information about the study and provided informed consent.

### Human Umbilical Cord-Derived Mesenchymal Stem Cell Isolation and Culture

Full-term-placentas with attached umbilical cords were obtained after standard deliveries at Thammasat Chalermprakiat Hospital. Cells obtained from the umbilical cord were produced as follows. The umbilical cord tissue was dissected into small fragments and subjected to digestion with 0.25% (w/v) trypsin-EDTA (GIBCO, Invitrogen Corporation, USA) for 30 min at 37 °C. The tissue fragments were rinsed twice with PBS and subsequently cultured in DMEM containing 10% (v/v) FBS (GIBCO, Invitrogen Corporation, USA) in a 25 cm^2^ culture flask (Corning, USA). The cells were subsequently grown at 37 °C. The culture medium was replaced every 3 days to remove nonadherent cells. The adherent cells were further cultured until fibroblast-like colonies were established. To promote cell expansion, cells were subcultured using 0.25% trypsin-EDTA. hUC-MSC morphology was examined using an inverted microscope (Nikon Eclipse Ts2R, Japan). Cell cultures were regularly monitored for growing fibroblast-like cell colonies.

### Isolation of Diarylheptanoid DPHD from *Curcuma comosa*

The diarylheptanoid (3*R*)-1,7-diphenyl-(4*E*,6*E*)-4,6-heptadien-3-ol (DPHD) was isolated from *Curcuma comosa* Roxb. following previously established protocols. In brief, the rhizomes were sliced, dried, pulverized, and extracted with *n*-hexane. After solvent evaporation, purification of diarylheptanoids from the hexane extract was performed using column chromatography. The chemical structure of ASPP 049 was characterized by NMR and MS, and its purity was determined to be greater than 98% using HPLC.^12^ The structure is shown in Figure 2A.

### Immunophenotypic Characterization of Human Umbilical Cord Mesenchymal Stem Cells via Flow Cytometry

Phenotypic characterization of hUC-MSCs was performed by flow cytometry (FACScalibur, Becton Dickinson, USA) with CellQuest software (Becton Dickinson, USA). Cells at passages 3–6 were harvested using 0.25% trypsin–EDTA and resuspended in phosphate-buffered saline (PBS). Cells were incubated with fluorochrome-conjugated mouse antihuman antibodies (CD45-FITC, CD34-PE, CD90-PE, CD73-PE; BioLegend, USA; CD105-PE; BD Biosciences, USA) for 30 min at 4 °C in the dark. After antibody incubation, the cells were washed twice with PBS and subsequently fixed in 1% (w/v) paraformaldehyde in PBS.

### The Multilineage Differentiation Assay

Human umbilical cord-derived MSCs (hUC-MSCs, passages 3–6) were subjected to osteogenic, adipogenic, and chondrogenic differentiation, using lineage-specific media. Cells were seeded in 6-well plates at 5 × 10^3^ cells/cm^2^ and maintained in DMEM with 10% FBS. For adipogenic differentiation, cultures at 70–80% confluence were switched to induction medium (high-glucose DMEM supplemented with 10% FBS and a cocktail of adipogenic agents—0.5 mM isobutylmethylxanthine, 1 μg/mL insulin, 100 nM dexamethasone, and 100 μM indomethacin (Sigma-Aldrich, USA)). The medium was replaced every 3 days, and after 28 days of induction, lipid accumulation was assessed using Oil Red O staining (Sigma-Aldrich, USA). To induce osteogenesis, cells at 80–90% confluence were maintained in osteogenic induction medium composed of low-glucose DMEM supplemented with 10% FBS, 100 nM dexamethasone, 10 mM β-glycerophosphate, and 50 μg/mL ascorbic acid; Sigma-Aldrich, USA) for 21 days with medium replacement every 3 days. Calcium accumulation was detected by Alizarin Red staining and subsequently examined with an inverted microscope (Nikon Eclipse Ts2R, Japan). For induction of chondrogenesis, hUC-MSCs were seeded in U-bottom 96-well plates at a density of 3 × 10^6^ cells/cm^2^ and incubated overnight at 37 °C under 5% CO_2_. Cultures were then maintained in ChondroMAX Differentiation Medium (Sigma-Aldrich, USA) with the culture medium replaced every 3 days. After 14 days, the resulting spheroid aggregates were fixed in 10% formalin, stained with 1% w/v Alcian Blue (Sigma-Aldrich, USA), and observed by inverted microscopy.

### Cell Viability and Proliferation Assays

Percent cell viability was calculated relative to untreated control cells. Cell viability was assessed using the MTT assay. Briefly, hUC-MSCs were seeded at a density of 1 × 10^3^ cells per well in 96-well plates (Costa, Corning, USA) and cultured in standard growth medium. After 24 h, cells were exposed to DPHD, dissolved in 0.05% DMSO, at final concentrations ranging from 1 to 50 μM, and incubated for 24–72 h. Control cells received culture medium containing the same concentration of methanol (0.05%) without DPHD. At each time point, cells were incubated with MTT solution (0.5 mg/mL) for 4 h. Following crystal formation, formazan crystals were dissolved in 150 μL of DMSO, and the absorbance was measured at 570 nm. Percent cell viability was determined relative to the untreated control group. To evaluate the growth kinetics, hUC-MSCs at passages 3–5 were seeded at a density of 5 × 10^2^ cells/cm^2^ in 24-well plates (Corning, USA) and maintained in complete medium. After 24 h, cells were treated with different doses of DPHD or 0.05% DMSO (control). Viable cells were quantified daily for 7 days using a hemocytometer. All measurements were performed in triplicate.

### Scratch Wound Healing Assay

MSCs were plated at a density of 4 × 10^4^ cells/well in 24-well plates. Following overnight incubation, a sterile 200 μL pipette tip was used to create a wound area by scratching the cell monolayer. Following the removal of debris, the culture was replenished with 500 μL of either 2.5 μM or 5 μM DPHD. The migration of MSCs into the wound area was observed using a phase contrast objective (10x) on an inverted microscope. The wound width was measured at 0, 6, 12, 24, 36, and 48 h over a 48-h period. The migration rate of MSCs was calculated by comparing the wound width at 0 h with that at subsequent time points, according to the following equation: Migration rate (%) = [(wound width at 0 h – wound width at time point)/wound width at 0 h] × 100.

### Wnt/β-Catenin Signaling Pathway Inhibition and DPHD Treatment

To investigate the involvement of Wnt/β-catenin signaling in both the proliferative and osteogenic effects of DPHD, hUC-MSCs were seeded into 6-well plates and cultured to approximately 70–80% confluence before undergoing pathway inhibition. Cells were pretreated with the Wnt/β-catenin inhibitor XAV939 (5 μM; Cayman Chemical, MI, USA) for 2 h in growth medium, after which the inhibitor-containing medium was removed and replaced with either fresh growth medium or osteogenic differentiation medium supplemented with DPHD (2.5 μM), depending on the experimental condition. For the proliferation assay, cells were incubated for an additional 24 h and subsequently harvested for RNA extraction to quantify Wnt/β-catenin–related genes, including *CTNNB1*, *CCND1*, and *C-MYC*. For the differentiation assay, cells were maintained in osteogenic medium containing DPHD for 14 days, with medium refreshed every 2–3 days, and harvested at the end of the induction period for analysis of osteogenic markers including *RUNX2*, *ALP*, and *COL1A1*.

### Senescence-Associated β-Galactosidase (β-Gal) Staining Assay

To confirm that hUC-MSCs undergo senescence during prolonged ex vivo expansion, cellular senescence was evaluated at different passages, including early (P3), middle (P5–8), and late passages (P13). Cells were seeded in 35 mm culture dishes at 2 × 10^5^ cells per dish and cultured for 5 days. At the end of culture, the medium was removed, and the dishes were rinsed twice with 1× PBS. Subsequently, 1 mL of fixative solution (1×) was added, and cells were fixed for 10–15 min at room temperature. The fixative was removed, and dishes were washed twice with 1× PBS to stop the fixation process. Thereafter, 1 mL of β-galactosidase staining solution (1×) was added, and the plates were sealed with parafilm to prevent evaporation. Cultures were incubated at 37 °C in a dry incubator without CO_2_, and the development of blue coloration was monitored under a light microscope, indicating senescence-associated β-galactosidase (SA-β-gal) activity. To further examine the effects of DPHD on cellular senescence, hUC-MSCs at passages P8 and P13 were treated with DPHD at final concentrations of 1, 2.5, 5, and 10 μM. After 5 days of treatment, cells were harvested and analyzed for SA-β-gal activity.

### Measurements of Reactive Oxygen Species

hUC-MSCs were seeded in black, clear-bottom 96-well plates at a density of 1 × 10^4^ cells per well and allowed to adhere overnight. Oxidative stress was induced by exposure to 400 μM H_2_O_2_ for 3 h. Following induction, cells were exposed to DPHD at final concentrations of 1, 2.5, or 5 μM and further incubated for 24 h. After treatment, cells were gently rinsed with PBS and incubated with 10 μM H_2_DCFDA for 45 min at 37 °C in the dark. Excess dye was subsequently removed by washing with PBS, and intracellular fluorescence was measured using an EnVision multimode plate reader. Relative intracellular reactive oxygen species (ROS) levels were quantified by normalizing fluorescence intensity to that of untreated control cells.

### Alkaline Phosphatase (ALP) Staining and Activity Assay

hUC-MSCs were plated in 6-well culture dishes at a density of 5 × 10^3^ cells/cm^2^ and maintained in osteogenic induction medium for 14 days. To evaluate alkaline phosphatase (ALP) activity by histochemical staining, the cultures were rinsed with PBS and fixed in 4% paraformaldehyde at 4 °C for 5 min. Following the addition of BCIP/NBT substrate (Sigma-Aldrich, USA), color development was allowed to proceed for 30 min at room temperature, and the reaction was subsequently terminated by washing with deionized water. Stained cells were visualized using a Nikon TS100 light microscope (Japan). For quantitative assessment of ALP activity, the SensoLyte pNPP Alkaline Phosphatase Assay Kit (Anaspec, USA) was employed. Following incubation of cell lysates with pNPP substrate for 30 min at room temperature, the reaction was terminated, and absorbance was measured at 405 nm. Enzyme activity was determined using a pNP standard curve and normalized to total protein content assessed by a BCA assay (Sigma-Aldrich, USA).

### Alizarin Red S Staining

To assess calcium deposition and matrix mineralization, osteoblast cultures were washed with PBS and fixed in 4% paraformaldehyde. Cells were then incubated with 2% Alizarin Red S for 20 min at room temperature to label calcium-rich nodules, after which the excess dye was removed by two rinses with deionized water. The stained mineralized areas were subsequently observed using a light microscope. Mineralization was quantified by extracting retained Alizarin Red S from stained cultures using 10% cetylpyridinium chloride (pH 7.0) for 30 min to solubilize the bound dye. The extracted solution was transferred to a 96-well plate, and absorbance was measured at 570 nm.

### Total RNA Isolation and Quantitative PCR of Genes

Total RNA was isolated using TRIzol reagent in accordance with the manufacturer’s instructions (Invitrogen, USA). RNA concentration and purity were assessed by measuring absorbance at 260 nm. For cDNA synthesis, 500 ng of total RNA was reverse-transcribed using the iScript Select cDNA Synthesis Kit (Bio-Rad Laboratories, USA). Quantitative real-time PCR (qRT-PCR) was performed using iTaq Universal SYBR Green Supermix (Bio-Rad Laboratories, USA) on an ABI StepOnePlus Real-Time PCR System (Applied Biosystems, Foster City, CA, USA). Relative gene expression levels were determined using the comparative cycle threshold (ΔCt) method, with GAPDH used as the endogenous reference gene. Primer sequences used for amplification are listed in Table 1.

**Table 1:** Primers for qRT-PCR

Gene	Accession number	Sequence (5′ to 3′)	Product length
*RUNX2*	NM_001369405.1	F-5′-CCTCGGAGAGGTACCAGATG-3′ R-5′-TTCCCGAGGTCCATCTACTG-3′	247
*ALP*	NM_001369805.2	F-5′-CCTTGCTCACTCACTCACTCC-3′ R-5′- TTTTTTTTGCCGTTCCAAAC-3′	182
*OCN*	NM_001199661.1	F-5′-GTGCAGAGTCCAGCAAAGGT-3′ R- 5′-TCAGCCAACTCGTCACAGTC-3′	175
*COL1A1*	NM_000088.4	F-5′-AGGGCCAAGACGAAGACATCCC-3′ R- 5′-TGTCGCAGACGCAGATCCG-3′	108
*C-MYC*	NM_012333.5	F-5′-ATGGCCCATTACAAAGCCG-3′ R-5′-TTTCTGGAGTAGCAGCTCCTAA-3′	175
*AXIN2*	NM_004655.4	F-5′-CCTGGCTCCAGAAGATCACA-3′ R-5′-AGCATCCTCCGGTATGGAAT-3′	120
*CCDN1*	NM_053056.3	F-5′-GATCAAGTGTGACCCGGACTG-3′ R-5′-CCTTGGGGTCCATGTTCTGC-3′	101
*CTNNB1*	NM_001438874.1	F-5′-GCTTGTTCGTGCACATCAGGA-3′ F-5′-TGTGAACATCCCGAGCTAGGA-3′	140
*NANOG*	NM_024865.4	F-5′-TTCCTTCCTCCATGGATCTG-3′ F-5′- TCTGCTGGAGGCTGAGGTAT-3′	213
*SOX2*	NM_003106.4	F-5′-ACACCAATCCCATCCACACT-3′ F-5′- GCAAACTTCCTGCAAAGCTC-3’	224
*OCT4*	NM_203289.6	F-5′-GAAGGATGTGGTCCGAGTGT-3′ F-5’- GTGAAGTGAGGGCTCCCATA-3′	183
*GAPDH*	NM_001357943.2	F-5′-GAGTCAACGGATTTGGTCGT-3′ R-5′-TTGATTTTGGAGGGATCTCG-3′	184

### Quantification of SASP-Associated Cytokines

The secretion of pro-inflammatory cytokines, including IL-1β, IL-6, and MCP-1, was assessed in hUC-MSCs at early (P3) and late (P13) passages using a multiplex bead-based flow cytometry assay (BioLegend, CA, USA). hUC-MSCs at passage 13 were treated with 1, 2.5, and 5 μM of DPHD. Following 5 days of treatment, culture supernatants were harvested and processed in accordance with the manufacturer’s protocol. Briefly, samples in 96-well plates were incubated with premixed capture beads and detection antibodies under shaking conditions for 2 h. Streptavidin-phycoerythrin was added and incubated for 30 min at 600 rpm at room temperature. Following incubation, the supernatants were carefully removed, and the beads were resuspended in wash buffer. The samples were transferred to fluorescence-activated cell sorting (FACS) tubes, and cytokine levels were quantified using a flow cytometry system (BD Biosciences, CA, USA). Data analysis was performed using LEGENDplex Data Analysis Software (BioLegend, CA, USA). Standard curves (0–200 ng/mL) were constructed for each cytokine, and final concentrations were adjusted to account for dilution factors.

### Statistical Analysis

Data are presented as mean ± standard error of the mean (SEM). Statistical significance was evaluated using one-way analysis of variance (ANOVA) followed by Newman–Keuls post hoc tests. A p-value <0.05 was considered statistically significant.

## Results

### Characterization of Umbilical Cord-Derived Human Mesenchymal Stem Cells

This study aimed to explore the biological effects of DPHD on human umbilical cord-derived mesenchymal stem cells (hUC-MSCs). To validate the reliability of subsequent experiments, the hUC-MSCs were first characterized based on the criteria defined by the International Society for Cellular Therapy (ISCT). The following section presents the characterization data and evaluations. In Figure 1A, primary cultures of hUC-MSCs adhered to plastic and exhibited a typical fibroblast-like, spindle-shaped morphology. Cells attached within 12 h, increased in density at 24–48 h, and by 120 h, formed a nearly confluent monolayer of homogeneous spindle-shaped cells. Trilineage differentiation was confirmed under osteogenic, adipogenic, and chondrogenic conditions. Osteogenic cultures exhibited extensive calcium deposition by Alizarin Red S staining, absent in undifferentiated controls. Adipogenic induction led to lipid droplet accumulation, as assessed by Oil Red O staining, with negligible staining in controls (undiff). Chondrogenic differentiation produced dense pellets with strong Alcian Blue staining, indicating glycosaminoglycan production, while controls remained unstained (Figure 1B). Flow cytometry confirmed the MSC phenotype with high expression of CD73 (99.82 ± 0.04%), CD90 (98.51 ± 0.87%), and CD105 (98.97 ± 0.08%), and minimal expression of hematopoietic lineage markers CD34 (0.9 ± 0.69%) and CD45 (0.59 ± 0.46%) (Figure 1C). Together, these results show that the isolated hUC-MSCs meet the minimal criteria of the International Society for Cellular Therapy (ISCT), exhibiting characteristic morphology, self-renewal, and trilineage differentiation potential.

**Figure 1:**
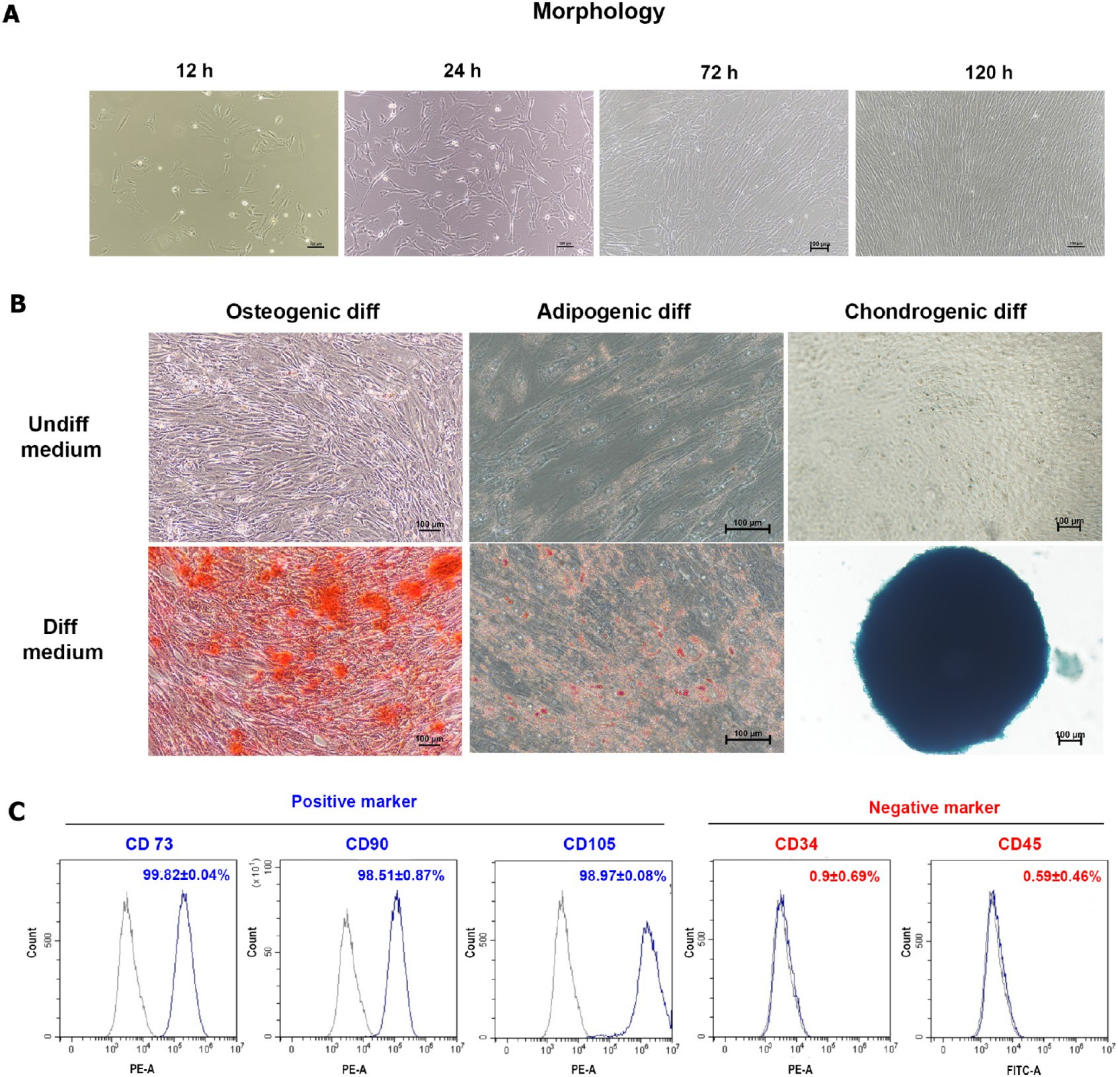
Comprehensive characterization of human umbilical cord-derived mesenchymal stem cells (hUC-MSCs). (A) Morphology of hUC-MSCs at 12, 24, 72, and 120 h after plating. Scale bar = 100 μm. (B) Multilineage differentiation potential. Lineage-specific differentiation into osteogenic, adipogenic, and chondrogenic phenotypes was validated by positive staining with Alizarin Red S, Oil Red O, and Alcian Blue, respectively, compared to undifferentiated controls. Scale bar = 100 μm. (C) Phenotypic analysis by flow cytometry revealed that MSCs were positive for CD73, CD90, and CD105, but negative for hematopoietic markers CD34 and CD45.

### Effect of DPHD on hUC-MSC Cell Viability, Proliferation, and Phenotype Stability

The proliferative response of hUC-MSCs to DPHD was evaluated by MTT assay. As shown in Figure 2B, DPHD at 1–5 μM significantly enhanced cell viability compared to controls, whereas higher concentrations (20–50 μM) elicited dose- and time-dependent cytotoxic effects. Consistent with these findings, growth curve analysis demonstrated that DPHD at 2.5 and 5 μM markedly enhanced cumulative cell numbers over a 7-day culture period (Figure 2C), confirming its growth-promoting effect. Conversely, cell proliferation was suppressed at higher concentrations, particularly at 20 μM.

**Figure 2:**
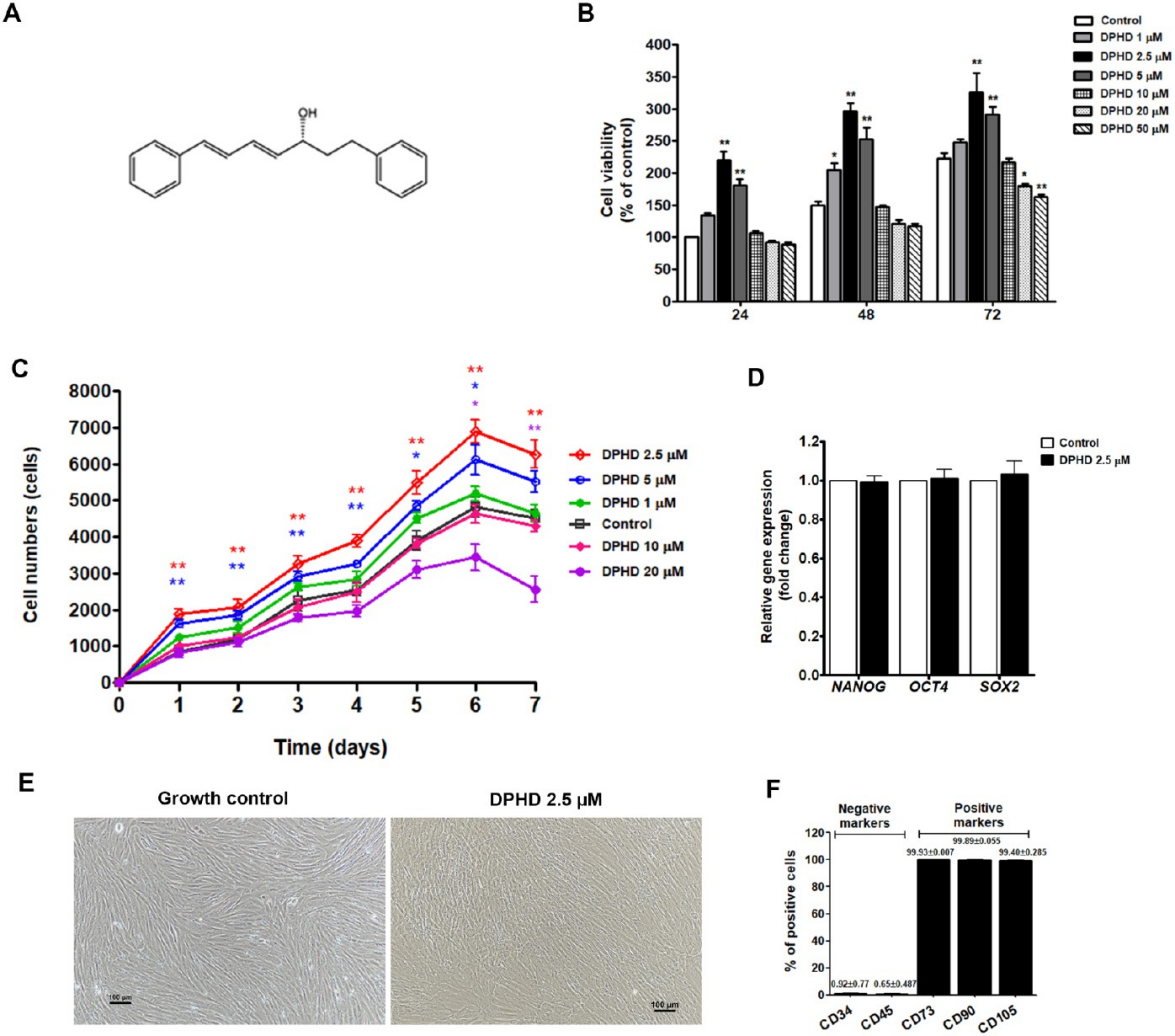
DPHD regulates cell viability, proliferative capacity, and phenotype of hUC-MSCs. (A) Chemical structure of DPHD. (B) Cell viability following 24, 48, and 72 h exposure to increasing concentrations of DPHD (1–50 μM). (C) Proliferation assay showing cell growth curves over 7 days under DPHD treatment (1–20 μM). Data are expressed as mean ± SEM *p < 0.05, **p < 0.01 compared with the control group at each time point. (D) mRNA expression of *NANOG*, *OCT4*, and *SOX2* after 5 days of treatment with 2.5 μM DPHD. GAPDH was used as the internal control. Values are presented as mean ± SEM (*n* = 3). (E) Representative morphology of hUC-MSCs after 5 days of treatment with 2.5 μM DPHD. (F) Flow cytometric analysis of MSC markers CD73, CD90, CD105 after 5-day treatment with 2.5 μM DPHD. Bars represent % positive cells (mean ± SEM, *n* = 3).

Importantly, despite enhanced proliferation, DPHD-treated hUC-MSCs retained their stem cell characteristics. To assess whether DPHD influences the stemness of UC-MSC, the mRNA expressions of *NANOG*, *OCT4*, and *SOX2* were determined. As shown in Figure 2D, no statistically significant differences were observed between the control and DPHD-treated (2.5 μM) groups after 5 days of treatment. Moreover, the cells retained mesenchymal stem cell characteristics, both in their morphology and in the expression of phenotypic surface markers (Figure 2E and F). These results demonstrate that DPHD not only enhances UC-MSC expansion but also preserves their stemness phenotype under optimal culture conditions.

### Effect of DPHD on Cell Migration and Wnt/β-Catenin Signaling Pathway

Cell migration is a critical process for tissue regeneration and wound healing, as it enables cells to move toward the injury site and initiate repair. To determine whether DPHD influences this regenerative property, a scratch wound healing assay was performed. Representative images at 0, 12, 24, 36, and 48 h are shown in Figure 3A. DPHD treatment markedly accelerated wound closure compared to the control. At 48 h, cells treated with 2.5 μM DPHD exhibited the greatest wound closure, followed by 5 μM DPHD. Quantitative analysis confirmed that DPHD significantly enhanced wound closure at all time points after 12 h, indicating that DPHD promotes cell motility and proliferation.

**Figure 3:**
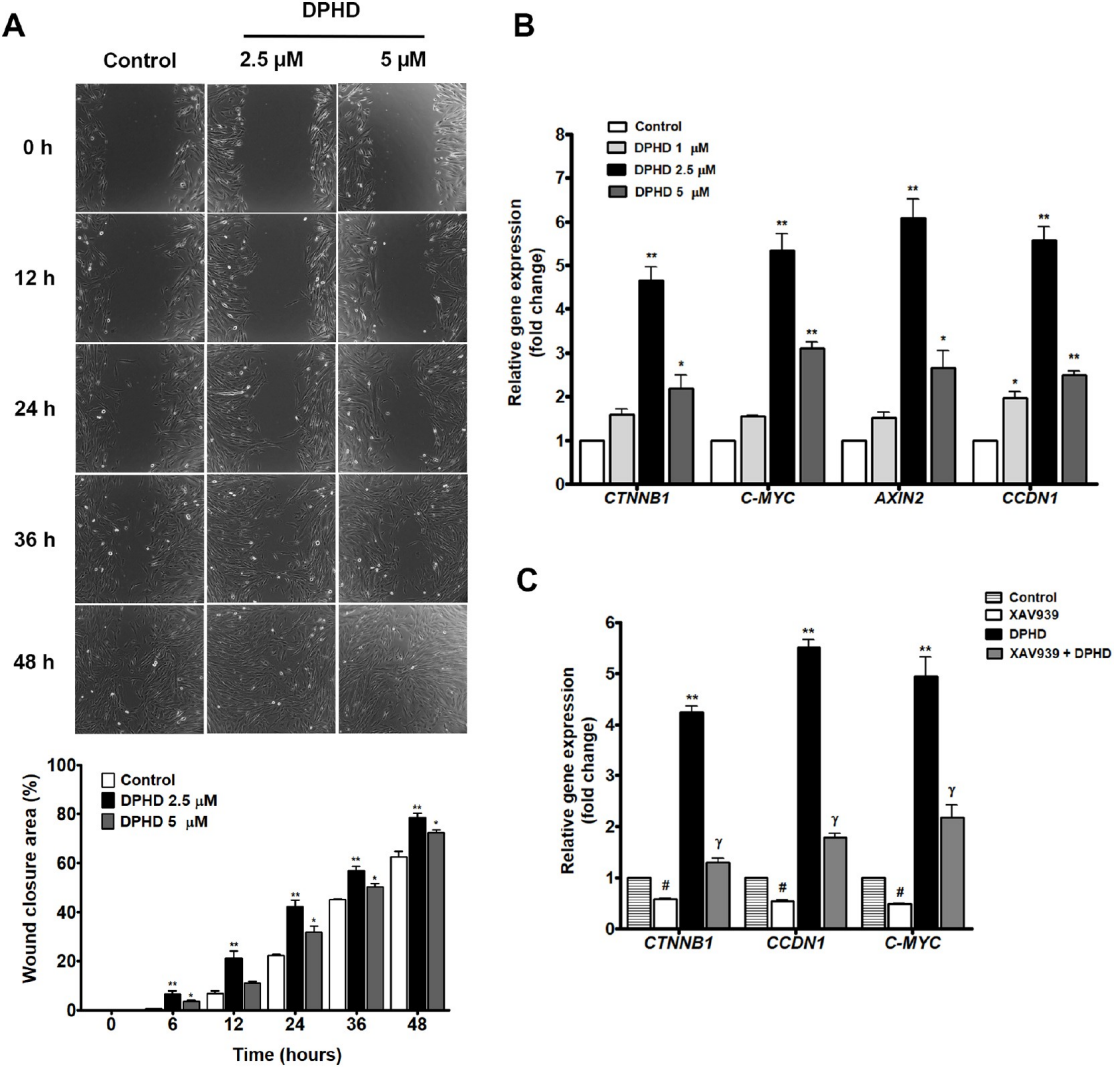
Effect of DPHD on cell migration and Wnt/β-catenin signaling. (A) Representative wound closure images at 0, 12, 24, 36, and 48 h in control and DPHD-treated cells (2.5 and 5 μM) from scratch assays. Quantitative analysis of wound closure (%) over time is shown as mean ± SEM (*n* = 3). *p < 0.05, **p < 0.01 compared with control. (B) Relative expression of Wnt/β-catenin-associated genes (*CTNNB1*, *C-MYC*, *AXIN2*, and *CCND1*) following 24 h DPHD treatment (1–5 μM). Values are expressed as mean ± SEM (*n* = 3). *p < 0.05, **p < 0.01 compared with the control group. (C) Inhibition of the Wnt/β-catenin pathway modulates DPHD-induced gene expression. Relative mRNA levels of *CTNNB1*, *CCND1*, and *C-MYC* were determined in cells treated with 2.5 μM DPHD, XAV939, and XAV939 combined with 2.5 μM DPHD. GAPDH was used as the internal control. Data are presented as fold change relative to control (mean ± SEM, *n* = 3). #*p* < 0.05 compared to the control group; γ*p* < 0.05, XAV939 + DPHD compared to the DPHD-treated group.

Since Wnt/β-catenin signaling is a key regulator of cell proliferation and osteogenesis, we next examined whether DPHD exerts its effects through activation of this pathway. To elucidate the underlying molecular mechanism, we analyzed the expression of Wnt/β-catenin downstream targets. As shown in Figure 3B, DPHD significantly upregulated the expression of *CTNNB1* (β-catenin), *C-MYC*, *AXIN2*, and *CCND1* after 24 h, suggesting that its pro-proliferative effect is mediated, at least in part, by activation of the canonical Wnt/β-catenin signaling pathway.

To further elucidate the role of Wnt/β-catenin signaling, cells were cotreated with DPHD and XAV939, a selective inhibitor of this pathway. As shown in Figure 3C, XAV939 markedly reduced DPHD-induced upregulation of *CTNNB1*, *CCND1*, and *C-MYC* (γ, *p* < 0.05, XAV939+ DPHD vs DPHD), bringing expression levels closer to baseline. XAV939 alone also suppressed these genes (#, *p* < 0.05 vs control). Collectively, these findings demonstrate that DPHD-mediated effects are strongly associated with Wnt/β-catenin signaling.

### Effect of DPHD on Cellular Senescence and ROS Accumulation in hUC-MSCs

To assess the effect of DPHD on cellular senescence, hUC-MSCs at different passages were subjected to SA-β-gal staining. The number of senescent cells increased progressively with passaging, rising sharply at passage 8 (P8) and reaching a peak at passage 13 (P13) (Figure 4A). DPHD treatment reduced senescence-associated β-galactosidase activity in a dose-dependent manner, with 2.5 and 5 μM significantly lowering senescent cell numbers at both P8 and P13 compared to controls (Figure 4B and 4C). In parallel, the expression of senescence-related genes, *p16* and *p21*, was examined. Both genes were markedly upregulated in late-passage cells (P13) compared to early passage cells (P3). However, treatment with DPHD effectively reduced the expression levels of *p16* and *p21*, with the strongest suppression observed at 2.5 μM (Figure 4D and 4E).

**Figure 4:**
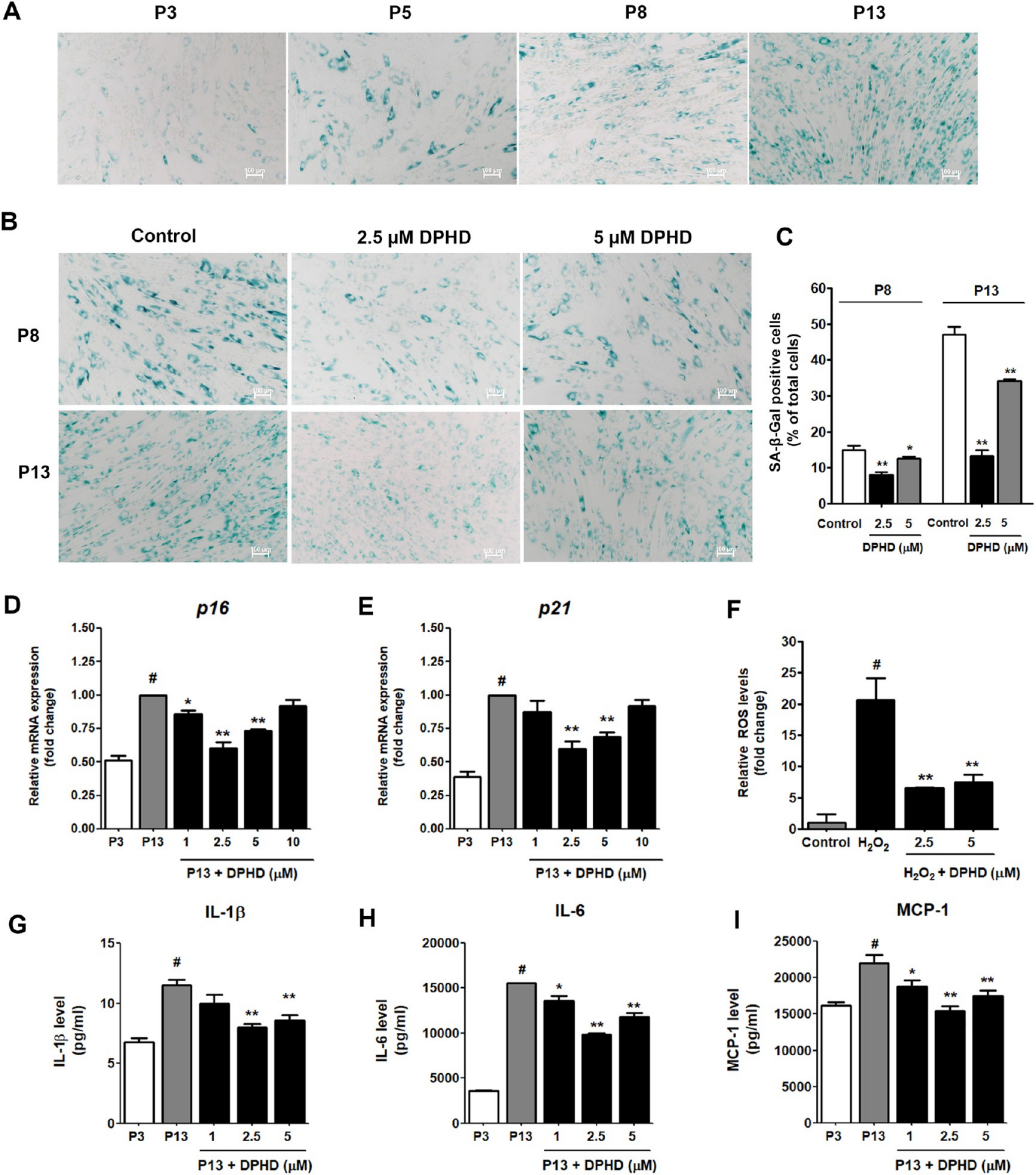
Effect of DPHD on hUC-MSC senescence. (A) SA-β-gal staining of hUC-MSCs at various passages (P), including P3, P5, P8, and P13. (B) Representative SA-β-gal staining images illustrating senescence in hUC-MSCs at P8 and P13 following treatment with DPHD (2.5 or 5 μM). (C) Quantification of SA-β-gal-positive cells after treatment with DPHD at P8 and P13 expressed as a percentage of total cells. (D–E) Relative expression of senescence-associated markers p16 (D) and p21 (E) was determined in P13 hUC-MSCs following DPHD treatment (1–10 μM), using GAPDH as the reference gene. (F) Relative ROS levels in cells treated with H_2_O_2_ or cotreated with DPHD at 2.5 μM and 5 μM. #*p* < 0.05 compared to the control. **p < 0.01 vs H_2_O_2_. Data are expressed as fold change relative to control (mean ± SEM, *n* = 3). (G–I) Pro-inflammatory cytokine levels; IL-1β, IL-6 and MCP-1 were measured using multiplex bead-based flow cytometry. Data are expressed as mean ± SEM *p < 0.05, **p < 0.01 vs P13 control; #*p* < 0.05 vs P3.

In addition to cell cycle regulators, senescence-associated secretory phenotype (SASP) factors were also examined. Late-passage hUC-MSCs exhibited elevated secretion of IL-1β, IL-6, and MCP-1 compared with early passage cells. DPHD supplementation effectively reduced the levels of these pro-inflammatory cytokines, with the strongest suppression observed at 2.5 and 5 μM (Figure 4G–I). These results indicate that DPHD attenuates cellular senescence in hUC-MSCs by reducing SA-β-gal activity, downregulating *p16* and *p21*, and suppressing pro-inflammatory SASP factors.

Since oxidative stress is a major contributor to cellular senescence, we next assessed whether DPHD could mitigate ROS accumulation under stress conditions. To evaluate the antioxidant property of DPHD, intracellular ROS levels were measured following H_2_O_2_ induced oxidative stress. As shown in Figure 4F, treatment with H_2_O_2_ markedly increased ROS levels by more than 20-fold compared to the control group (#*p* < 0.05). Co-treatment H_2_O_2_ with 2.5 μM and 5 μM DPHD significantly attenuated the H_2_O_2_ induced increase in ROS levels, indicating that DPHD possesses antioxidant activity. These findings suggest that DPHD may help preserve cellular function and delay senescence by reducing oxidative stress.

### Effect of DPHD on hUC-MSC Cell Cycle

Cell cycle analysis revealed clear differences between early- and late-passage hUC-MSCs. At P3, most cells were in G0/G1, with fewer in S and G2/M phases. By P13, cells showed pronounced G0/G1 arrest with reduced S and G2/M populations, consistent with replicative senescence (Figures 5A and 5B). DPHD treatment reversed these changes: treatment with 1, 2.5, and 5 μM DPHD significantly decreased the accumulation of cells in the G0/G1 phase, while increasing the proportions of cells in the S and G2/M phases compared to untreated P13 controls. These results indicate that DPHD alleviates G1/G0 arrest and promotes cell cycle progression in senescent hUC-MSCs.

**Figure 5:**
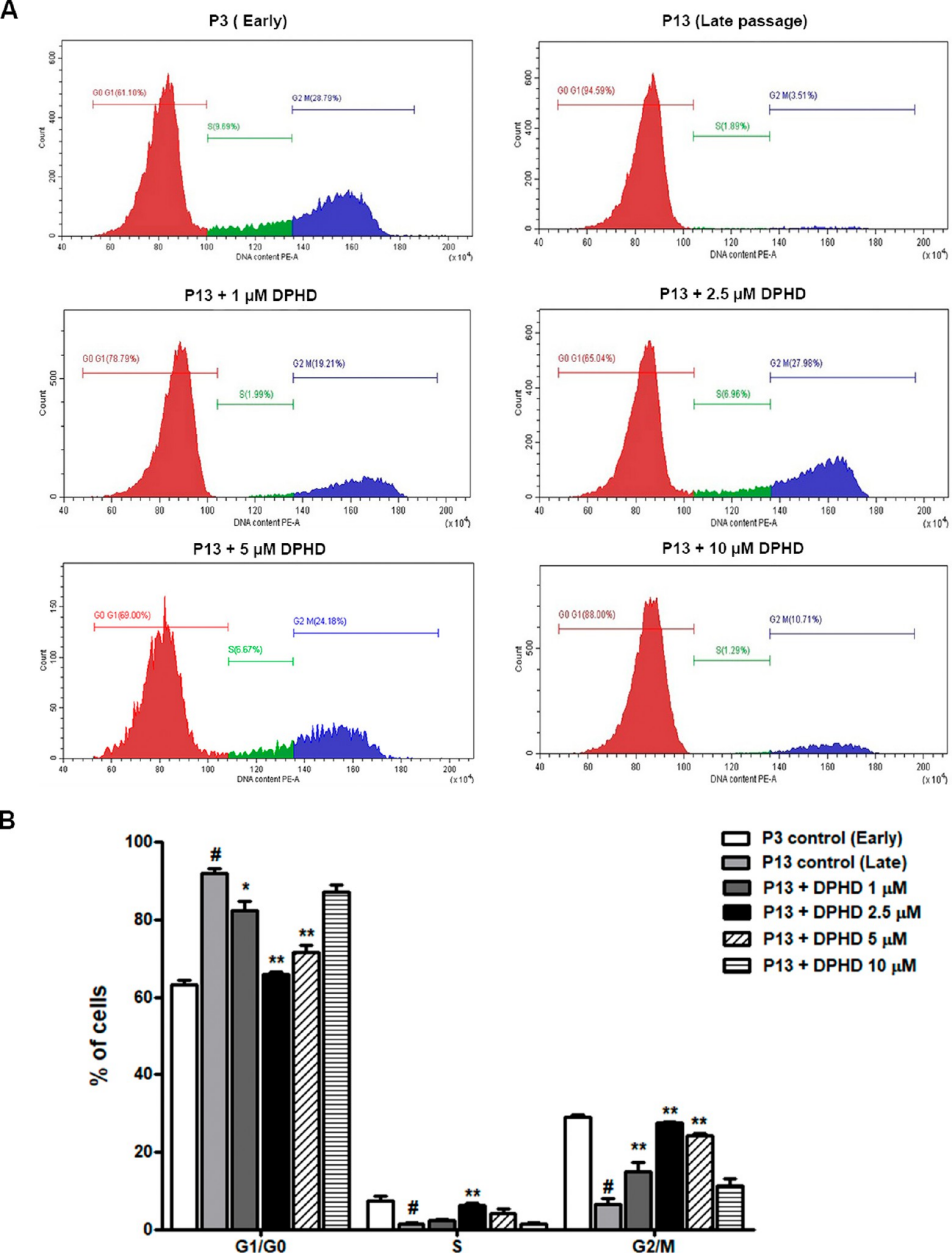
Effect of DPHD on cell cycle distribution of hUC-MSCs. (A) Representative flow cytometry profiles showing cell cycle distribution in early passage (P3) and late-passage (P13) hUC-MSCs, as well as P13 cells treated with DPHD (1–10 μM) for 48 h. (B) Quantification of cell cycle phases. Data are presented as mean ± SEM. #*p* < 0.05 vs P3 (early passage control); **p < 0.01 vs P13 control.

### DPHD Promoted Osteogenic Differentiation

To determine the role of DPHD in regulating osteogenic differentiation, hUC-MSCs were cultured under osteogenic conditions in the presence or absence of DPHD. As shown in Figure 6A, undifferentiated cells exhibited no alkaline phosphatase (ALP) or Alizarin Red S staining. In contrast, cells in the osteogenic control group showed positive ALP expression at day 14 and calcium deposition at day 21. Notably, supplementation with DPHD (1, 2.5, and 5 μM) enhanced osteogenic differentiation, as evidenced by more intense ALP and Alizarin Red S staining compared to the differentiation control. Quantitative analysis confirmed that DPHD significantly increased ALP activity at day 14 and calcium mineralization at day 21, with the most pronounced effects observed at 2.5 and 5 μM (Figures 6B and C). These findings suggest that DPHD promotes the osteogenic differentiation of hUC-MSCs.

**Figure 6:**
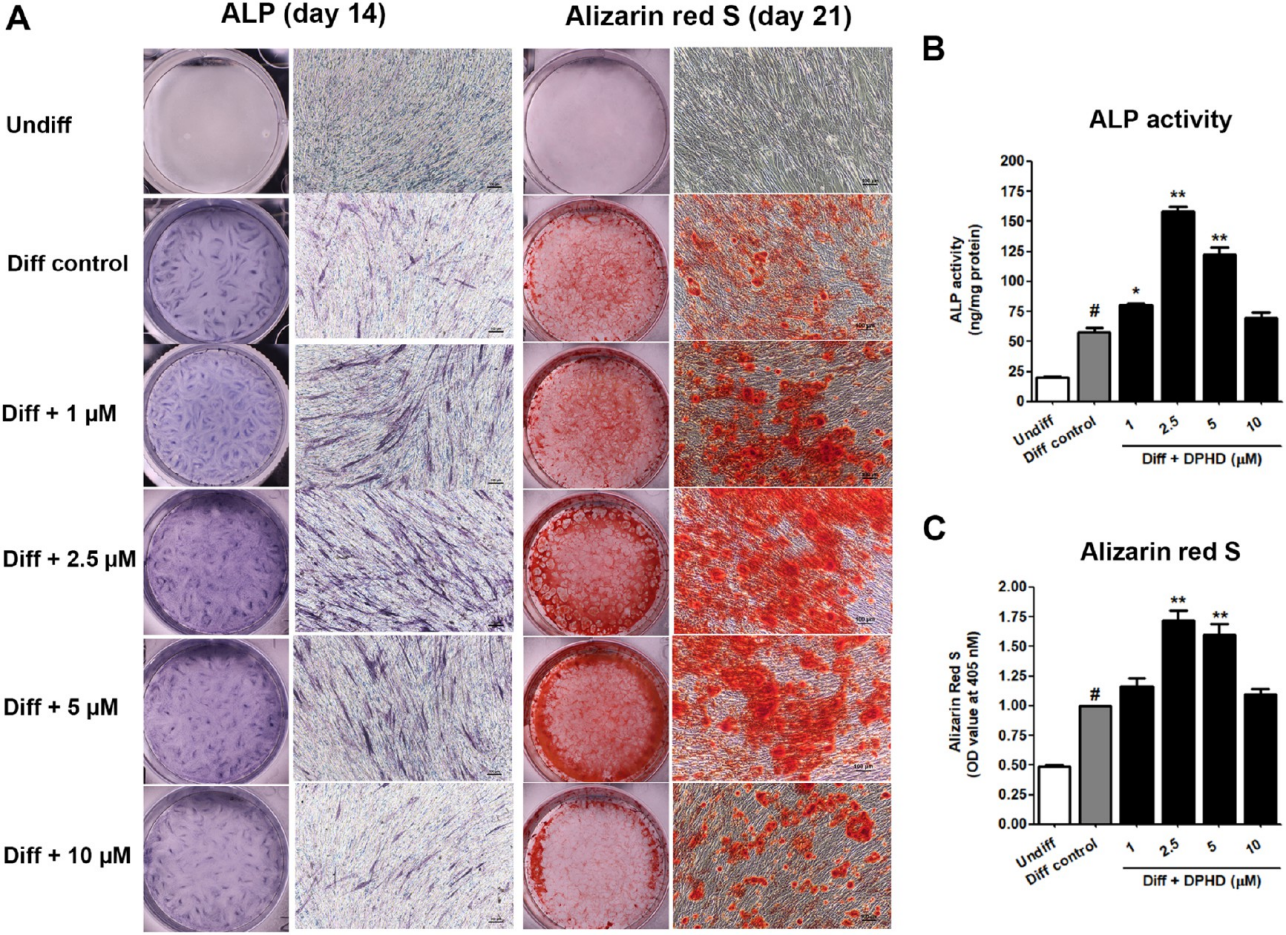
DPHD promotes osteogenic differentiation of hUC-MSCs. (A) Representative images of ALP staining at day 14 and Alizarin Red S staining at day 21 in undifferentiated (Undiff), differentiation control, and DPHD-treated groups (1–10 μM). (B) Measurement of ALP activity. (C) Quantitative assessment of Alizarin Red S staining. Values represent mean ± SEM. #*p* < 0.05 relative to the undifferentiated group; *p < 0.05, **p < 0.01 relative to the differentiation control.

### DHPD-Induced Osteogenic Gene Expression is Mediated through the Wnt/β-Catenin Pathway

To further evaluate the osteogenic effect of DPHD, the expression of osteoblast marker genes was analyzed by qRT-PCR at 7 and 21 days of differentiation. As shown in Figure 7A, DPHD significantly upregulated the expression of early and late osteogenic markers in a dose- and time-dependent manner. *RUNX2* and *ALP*, representing early osteoblast differentiation, were markedly induced at both 7 and 21 days, with the strongest effect observed at 2.5 and 5 μM DPHD. Similarly, *COL1A1* and *OCN,* markers of matrix synthesis and late-stage mineralization, were substantially elevated at 21 days under DPHD treatment. The induction was most pronounced at 2.5 μM DPHD, followed by 5 μM, while 10 μM exhibited a weaker effect. These results indicate that DPHD enhances osteoblast differentiation by modulating key osteogenic transcriptional programs.

**Figure 7:**
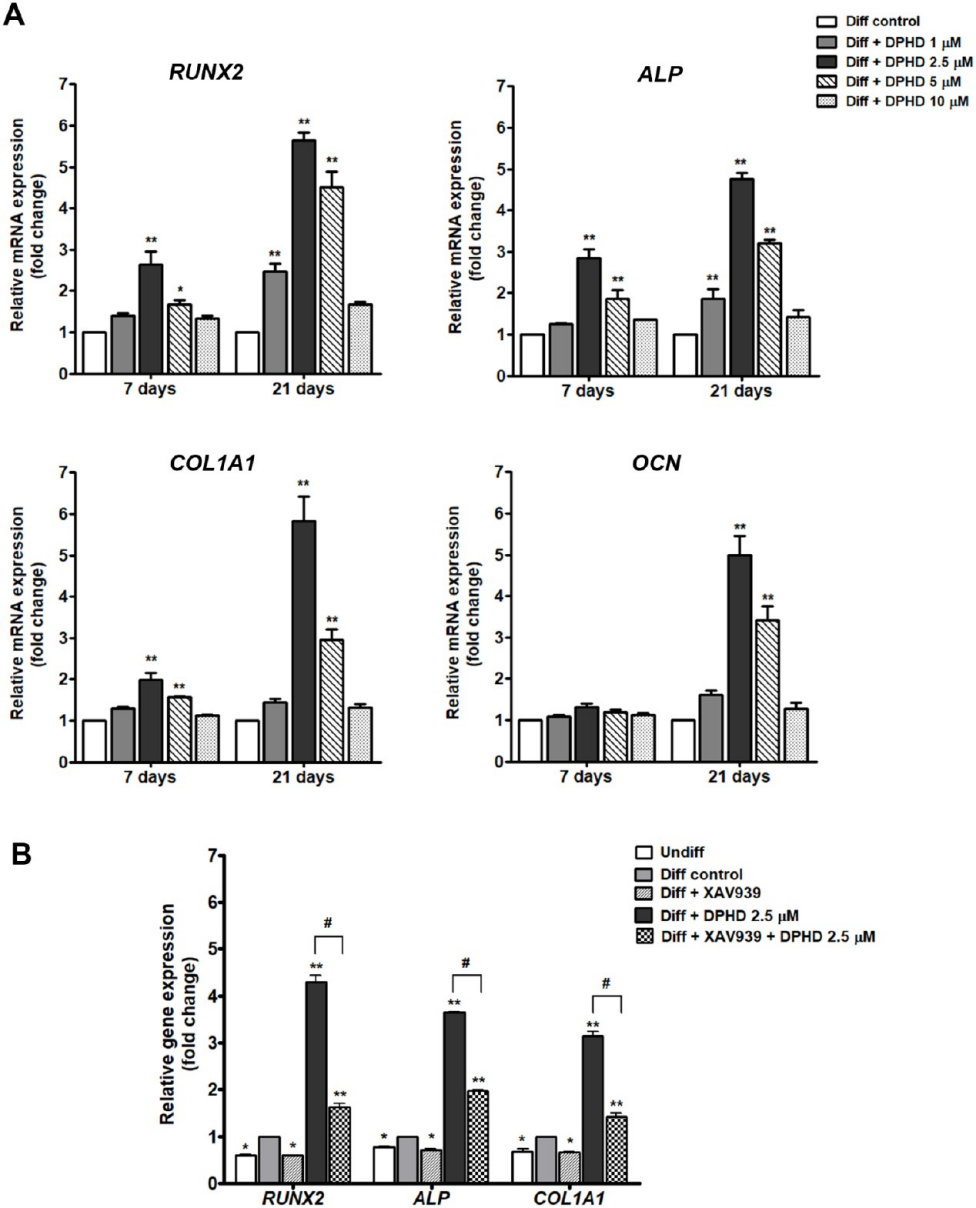
Effects of DHPD on osteogenic gene expression during differentiation. (A) Relative mRNA expression of *RUNX2*, *ALP*, *COL1A1*, and *OCN* in hUC-MSCs during osteogenic induction with or without DPHD (1–10 μM) for 7 and 21 days. GAPDH served as the internal reference gene. Data are expressed as mean ± SEM. *p < 0.05, **p < 0.01 compared with the differentiation control. (B) Relative mRNA expression of *RUNX2*, *ALP*, and *COL1A1* in undifferentiated cells (Undiff), differentiation control (Diff control), Diff + XAV939 (Wnt/β-catenin inhibitor), Diff + DHPD (2.5 μM), and Diff + XAV939 + DHPD for 14 days. Data are expressed as mean ± SEM. *p < 0.05, **p < 0.01 compared with the differentiation control; #*p* < 0.05 comparison between DPDH-treated and DPDH + XAV939 treated groups.

To elucidate whether Wnt/β-catenin signaling contributes to the osteogenic effects of DHPD, cells were cotreated with XAV939, a Wnt pathway inhibitor (Figure 7B). The DHPD-induced increase in *RUNX2*, *ALP*, and *COL1A1* mRNA expression was markedly suppressed by XAV939. These findings indicate that activation of the Wnt/β-catenin pathway is at least partially required for DHPD-mediated osteogenic gene expression.

## Discussion

In this study, we examined the biological effects of DPHD on hUC-MSCs, focusing on viability, senescence, cell cycle regulation, migration, antioxidant activity, and osteogenic differentiation. Our findings demonstrate that DPHD exerts a dual beneficial role by alleviating replicative senescence during prolonged in vitro expansion and enhancing osteogenic lineage commitment, while preserving stemness and promoting cell migration. Mechanistically, DPHD upregulates Wnt/β-catenin signaling, supporting its role in proliferation and differentiation. These results highlight DPHD as a promising natural small molecule for improving MSC-based regenerative medicine and addressing limitations in current therapies for bone-related disorders.

Consistent with the minimal criteria for defining MSCs,^18^ our isolated hUC-MSCs exhibited fibroblast-like morphology, expressed characteristic surface markers (CD73, CD90, CD105), and demonstrated trilineage differentiation capacity. Importantly, exposure to DPHD did not alter this immunophenotypic profile, confirming that stem cell identity was preserved even after treatment. In addition, DPHD treatment maintained the expression of key stemness markers (NANOG, OCT4, and SOX2), suggesting that DPHD not only preserves MSC identity but may also support pluripotency-associated transcriptional programs during extended culture. This observation is critical for translational applications, since loss of stemness and phenotypic drift during in vitro expansion remain major obstacles to MSC-based therapy.^19^

Our study revealed that DPHD exerts potent antisenescence effects in hUC-MSCs, as evidenced by reduced SA-β-gal-positive cells and downregulation of *p16* and *p21*, indicating delayed cell cycle arrest and sustained proliferative capacity.^20^ Moreover, the suppression of SASP-associated cytokines, including IL-1β, IL-6, and MCP-1, suggests that DPHD not only regulates intrinsic senescence pathways but also modulates the pro-inflammatory microenvironment created by senescent cells.^21,22^ Given that SASP factors reinforce senescence, impair tissue regeneration, and contribute to age-related inflammation, their suppression by DPHD may help preserve both the stemness and differentiation potential of hUC-MSCs during long-term culture. Flow cytometric analyses further confirmed that DPHD alleviated G0/G1 arrest and increased the proportion of cells in S and G2/M phases, underscoring its role in sustaining proliferative activity. By maintaining cell cycle progression, DPHD may extend the proliferative lifespan of MSCs and improve yields for therapeutic use. Furthermore, DPHD significantly attenuated H_2_O_2_-induced ROS accumulation, confirming its antioxidant capacity. By mitigating oxidative stress, DPHD may further contribute to delaying senescence and preserving MSC functionality. These effects are in line with previous findings on other phytochemicals, such as resveratrol and curcumin which have been shown to delay cellular senescence and enhance MSC functionality by modulating oxidative stress and cell cycle pathways.^23,24^

In addition to delaying senescence, DPHD significantly promoted osteogenic differentiation. These findings were demonstrated by increased ALP activity, enhanced calcium deposition, and elevated expression of key osteogenic genes, including *RUNX2*, *ALP*, *COL1A1*, and *OCN.* Notably, RUNX2, a master regulator of osteoblast differentiation, was strongly induced, indicating that DPHD acts at the early commitment stage of osteogenesis.^25^ Furthermore, sustained expression of *COL1A1* and *OCN* suggested that DPHD also promotes late-stage osteoblast maturation and mineralization.^26^ Importantly, the observed enhancement of osteogenesis is likely a downstream effect of senescence alleviation, as senescent mesenchymal stem cells (MSCs) are known to exhibit impaired differentiation capacity.

The translational relevance of these findings lies in the potential application of DPHD in bone-related disorders. Current pharmacological treatments for osteoporosis, fracture nonunion, and critical-size bone defects primarily regulate bone turnover but do not restore regenerative capacity.^27,28^ By promoting both the expansion and osteogenic differentiation of MSCs, DPHD may serve as a valuable adjunct to stem cell-based therapy for enhanced bone regeneration. Although the mechanistic pathways were not directly investigated, it is plausible that DPHD acts through key signaling cascades governing proliferation and osteogenesis, such as the Wnt/β-catenin pathway.^29^ The Wnt/β-catenin pathway is a critical signaling pathway that promotes cell proliferation and osteogenesis by activating downstream genes that regulate bone cell differentiation and growth.^30^ The observed upregulation of proliferation-related genes, including *CTNNB1*, *CCDN1*, *AXIN2* and *C-MYC*, is consistent with potential involvement of this signaling cascade.^31^ Importantly, the reversal of these effects by XAV939, a Wnt pathway inhibitor, supports the hypothesis that DPHD acts primarily via Wnt pathway activation rather than through alternative mechanisms. The increased expression of Cyclin D1 and c-Myc further highlights its potential role in regulating proliferative and survival pathways. In addition to these molecular changes, DPHD enhanced cell migration as demonstrated by scratch wound healing assays, which is critical for tissue repair and regenerative processes. This pro-migratory effect may also be mediated through Wnt/β-catenin activation, as Wnt signaling is known to regulate cytoskeletal dynamics and motility in mesenchymal stem cells. Notably, previous studies have reported that DPHD derived from *C. comosa* promotes osteoblast proliferation and differentiation via ERα-, Akt-, and GSK-3β-mediated activation of β-catenin signaling, providing additional support for the involvement of this pathway.^16^ Activation of this pathway promotes nuclear accumulation of β-catenin, which subsequently interacts with TCF/LEF transcription factors to drive the expression of osteogenic regulators such as RUNX2.^32^ RUNX2, in particular, is a master transcription factor that initiates osteoblast commitment, while downstream targets such as ALP, COL1A1, and OCN mediate matrix maturation and mineralization.^33,34^ Consistent with this canonical pathway, DPHD-treated hUC-MSCs exhibited significant upregulation of *RUNX2*, *ALP*, *COL1A1*, and *OCN*. Importantly, the present study further demonstrates that the osteogenic effects of DPHD are at least partially dependent on Wnt/β-catenin signaling, as cotreatment with the Wnt inhibitor XAV939 markedly suppressed DPHD-induced *RUNX2*, *ALP*, and *COL1A1* expression. These findings strengthen the evidence that DPHD enhances osteogenic differentiation through activation of the Wnt/β-catenin axis. Future mechanistic studies are needed to confirm this signaling axis and identify the direct molecular targets of DPHD.

This study has limitations. All experiments were performed in vitro; thus, in vivo validation is required to establish whether DPHD-treated MSCs retain improved engraftment, survival, and regenerative capacity after transplantation. Furthermore, the safety profile and potential off-target effects of DPHD need to be evaluated before translation into clinical settings. In summary, DPHD alleviates senescence, sustains proliferative activity, enhances osteogenic differentiation, maintains stemness, promotes cell migration, and exhibits antioxidant activity in hUC-MSCs. These properties position DPHD within the class of rejuvenating phytochemicals with potential translational value. Further in vivo and clinical studies are warranted to explore its utility in bone regeneration and age-related degenerative conditions.

## Conclusions

This report is the first to demonstrate that DPHD enhances the functional properties of hUC-MSCs by enhancing proliferative capacity, delaying cellular senescence, and potentiating osteogenic differentiation. These findings support DPHD as a promising agent for promoting the expansion and osteogenic induction of hUC-MSCs, highlighting its potential application in stem cell-based therapies, particularly in bone regenerative medicine.

## Supplementary Material



## Data Availability

Data sets and analyses from this study can be provided by the corresponding author upon reasonable request. All data in this study are presented in the manuscript.

## Notes

^1^H NMR spectrum of (3R)-1,7-diphenyl-(4E,6E)-4,6-heptadien-3-ol (DPHD) (400 MHz, CDCl_3_) (Figure S1); ^13^C NMR spectrum of (3R)-1,7-diphenyl-(4E,6E)-4,6-heptadien-3-ol (DPHD) (100 MHz, CDCl_3_) (Figure S2); ESI-TOF-MS of (3R)-1,7-diphenyl-(4E,6E)-4,6-heptadien-3-ol (ASDT001) (Figure S2) (PDF)

## Abbreviations

DPHD: (3R)-1,7-Diphenyl-(4E,6E)-4,6-heptadien-3-ol
ALP: Alkaline phosphatase
ARS: Alizarin Red S
BM-MSCs: Bone marrow-derived MSCs
COL1A1: Collagen type I alpha 1 chain
DMSO: Dimethyl sulfoxide
FBS: Fetal bovine serum
FITC: Fluorescein isothiocyanate
MSCs: Mesenchymal stem cells
MTT: 3-(4,5-Dimethylthiazol-2-yl)-2,5-diphenyl tetrazolium bromide
RUNX2: Runt-related transcription factor 2
ROS: Reactive oxygen species
SASP: Senescence-associated secretory phenotype
hUC-MSCs: Human umbilical cord-derived mesenchymal stem cells
