# Supplementary material for: DPHD from Curcuma comosa Enhances the Expansion and Osteogenic Differentiation of Human Umbilical Cord-Derived Mesenchymal Stem Cells While Attenuating Senescence
Source: ACS Omega. 2026 May 18;11(21):30361–74. doi: 10.1021/acsomega.5c08638 (PMC13234631; doi:10.1021/acsomega.5c08638)
Supplement: Supplementary file 1 [file ao5c08638_si_001.pdf]

Supporting Information

DPHD from *Curcuma comosa* Enhances the Expansion and Osteogenic Differentiation of Human Umbilical Cord-Derived Mesenchymal Stem Cells While Attenuating Senescence

Nittaya Boonmuen[¥], Moe Moe Paing[ψ], Nareerat Sutjarit[†], Pakpoom Kheolamai[ψ,γ], Sirikul Manochantr[ψ,γ], Chairat Tantrawatpan[ψ,γ], Waraluck Chaichompoo[#], Apichart Suksamrarn[#], Duangrat Tantikanlayaporn[ψ,γ]*

[¥]Department of Physiology, Faculty of Science, Mahidol University, Bangkok 10400, Thailand

[ψ]Center of Excellence in Stem Research and Innovation, Faculty of Medicine, Thammasat University, Pathum Thani 12120, Thailand

[†]Nutrition Unit, Faculty of Medicine Ramathibodi Hospital, Mahidol University, Bangkok 10400, Thailand

[γ]Division of Cell Biology, Faculty of Medicine, Thammasat University, Pathum Thani 12120, Thailand

[#]Department of Chemistry and Center of Excellence for Innovation in Chemistry, Faculty of Science, Ramkhamhaeng University, Bangkok 10240, Thailand

*Correspondence to:

Duangrat Tantikanlayaporn

Division of Cell Biology

and Center of Excellence in Stem Research and Innovation,

Faculty of Medicine, Thammasat University, Pathum Thani 12120, Thailand

E-mail address: dkanlayaporn@gmail.com, duangrat@staff.tu.ac.th

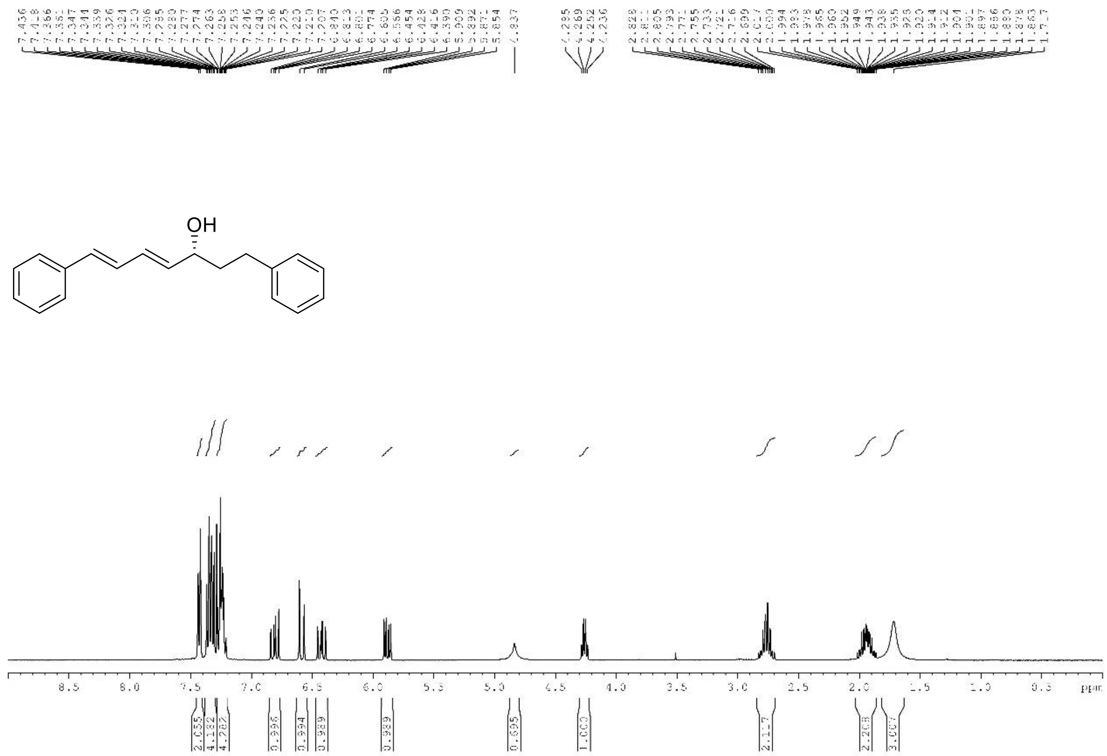

Figure S1. ^{1}H NMR spectrum of (3R)-1,7-diphenyl-(4E,6E)-4,6-heptadien-3-ol (DPHD) (400 MHz, $CDCl_3$)

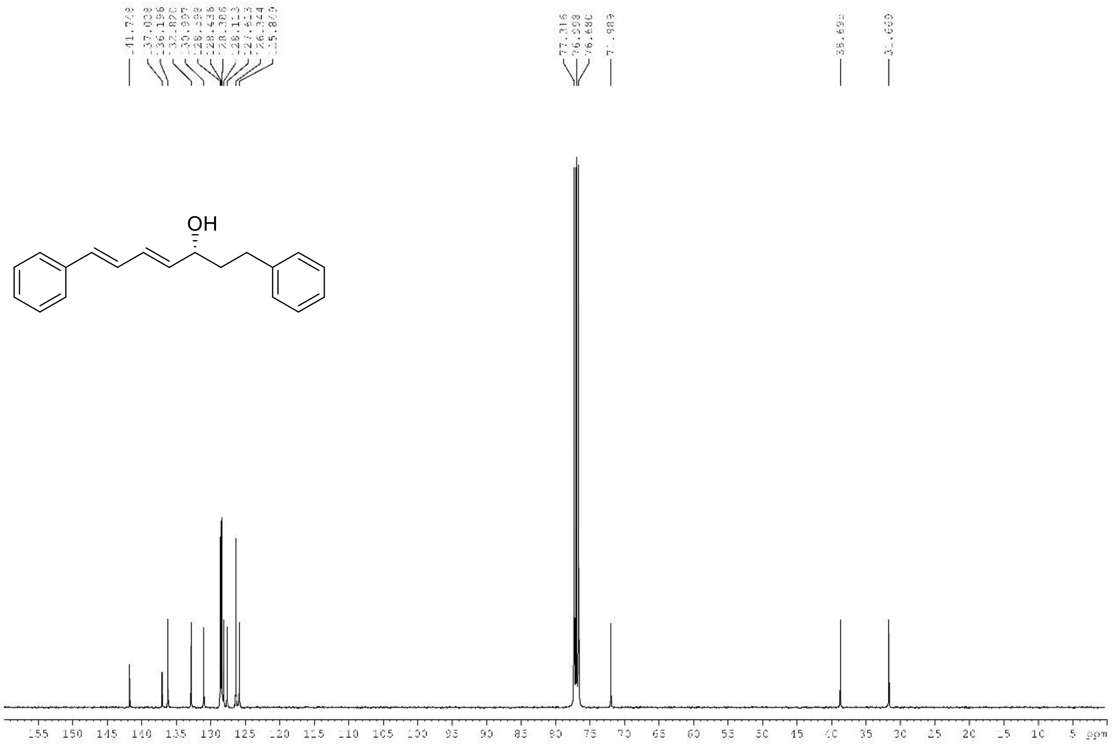

Figure S2. ^{13}C NMR spectrum of (3R)-1,7-diphenyl-(4E,6E)-4,6-heptadien-3-ol (DPHD) (100 MHz, $CDCl_3$)

Acquisition Parameter

Source Type	ESI	Ion Polarity	Positive	Set Nebulizer	2.0 Bar
Focus	Not active			Set Dry Heater	200 °C
Scan Begin	50 m/z	Set Capillary	4500 V	Set Dry Gas	8.0 l/min
Scan End	2000 m/z	Set End Plate Offset	-500 V	Set Divert Valve	Waste

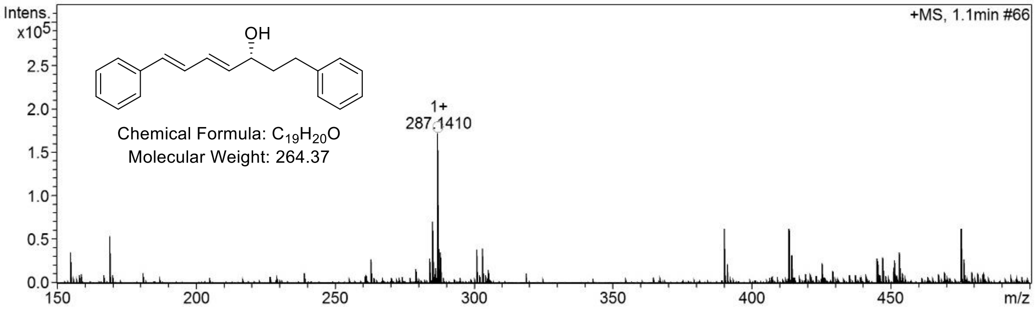

Meas. m/z	#	Ion Formula	m/z	|err| [mDa]	err [ppm]	Mean err [ppm]	rdb	N-Rule	e⁻ Conf	mSigma
287.140961	1	C19H20NaO	287.140636	0.3	1.1	-0.5	9.5	ok	even	8.7
	2	C17H21Na2O	287.138230	2.7	9.5	-8.9	6.5	ok	even	12.7
	3	C12H23N2Na4	287.144652	3.7	-12.9	13.3	0.5	ok	even	38.3

Figure S3. ESI-TOF-MS of (3R)-1,7-diphenyl-(4E,6E)-4,6-heptadien-3-ol (ASDT001)
